# Functional Richness and Identity Do Not Strongly Affect Invasibility of Constructed Dune Communities

**DOI:** 10.1371/journal.pone.0169243

**Published:** 2017-01-10

**Authors:** Tanya J. Mason, Kristine French, Dianne F. Jolley

**Affiliations:** 1 Centre for Sustainable Ecosystem Solutions, School of Biological Sciences, University of Wollongong, New South Wales, Australia; 2 School of Chemistry, University of Wollongong, New South Wales, Australia; University of Oregon, UNITED STATES

## Abstract

Biotic effects are often used to explain community structure and invasion resistance. We evaluated the contribution of functional richness and identity to invasion resistance and abiotic resource availability using a mesocosm experiment. We predicted that higher functional richness would confer greater invasion resistance through greater resource sequestration. We also predicted that niche pre-emption and invasion resistance would be higher in communities which included functional groups similar to the invader than communities where all functional groups were distinct from the invader. We constructed communities of different functional richness and identity but maintained constant species richness and numbers of individuals in the resident community. The constructed communities represented potential fore dune conditions following invader control activities along the Australian east coast. We then simulated an invasion event by bitou (*Chrysanthemoides monilifera* ssp. *rotundata* DC. Norl.), a South African shrub invader. We used the same bitou propagule pressure across all treatments and monitored invasion success and resource availability for 13 months. Contrary to our predictions, we found that functional richness did not mediate the number of bitou individuals or bitou cover and functional identity had little effect on invasion success: there was a trend for the grass single functional group treatment to supress bitou individuals, but this trend was obscured when grasses were in multi functional group treatments. We found that all constructed communities facilitated bitou establishment and suppressed bitou cover relative to unplanted mesocosms. Abiotic resource use was either similar among planted communities, or differences did not relate to invasion success (with the exception of light availability). We attribute invasion resistance to bulk plant biomass across planted treatments rather than their functional group arrangement.

## Introduction

Plant invasion may be predicted in part by stochastic events such as dispersal (e.g. [1]) or by historical contingency where prior species influence the success of deferred species (e.g. [2, 3], but see [4]). However, deterministic mechanisms within the community–namely biological interactions may also be important (e.g. [5, 6]). Understanding the contribution of biological interactions to invader success, community resistance and resource availability has important practical as well as theoretical ecological implications: if these interactions strongly contribute to invasion, they may be manipulated to achieve positive conservation and restoration outcomes.

In order to understand the role of biological interactions in invasion, researchers focused initially on invader attributes which contribute to invasion success such as high growth rates, efficient resource use or high resource allocation to reproduction (e.g. [7, 8]). More recently the biological role of the resident or invaded community has been recognised. Theoretical and modelling approaches, and to some extent, constructed community studies, often indicate that richer communities are more resistant to invasion than less speciose communities (e.g. [9, 10, 11]). This pattern of resistance may be attributed to a greater likelihood of strong competitors or resistant species being present in richer rather than simpler communities–the ‘sampling effect’ (e.g. [12, 13]). However, empirical studies (such as spatial pattern and species addition studies) often refute the belief that species diversity enhances invasion resistance (reviewed in [14]). Finally, interactions between the invader and invaded community are important in determining community structure. The environmental niche of both the invader and resident species determine the degree of niche overlap or complementarity exhibited in the community. If the invader occupies a different niche to the rest of the community, there may be resources available to allow invader establishment. If there is considerable niche overlap between the invader and resident species, competition may exclude or moderate invader biomass [15, 16, 17]. As an extension to the niche complementarity theory, it has also been proposed that if an invader is functionally similar to members of the resident community, then invasion will be less successful than if the invader is functionally distinct (e.g. [12, 16]).

Plant composition may have greater importance than richness in determining invasion outcomes. This idea has been established at the species level [18], and the importance of functional composition or identity has also been documented [19, 20]. The spatial and temporal partitioning of abiotic resources means that communities with different functional richness and composition values should have different resource availability: for example, taller growth forms intercept light before it reaches smaller growth forms, and groups with faster growth rates sequester nutrients before those with slower growth rates.

Much of the theoretical work examining biotic resistance–where resident communities reduce the success of invaders [21]—has considered interactions at the level of species identity. However, a focus on higher levels of biological organisation such as plant functional types is justified. In this typology, species are categorised based on similarity of function using traits such as those related to growth form [22]. Size and shape are often good indicators of competitive ability and resource acquisition [23], and growth form is relevant to ecosystem functions such as carbon assimilation [24]. The relevance of growth form as a basis for functional groups in our study system has been tested previously. In a competitive hierarchy experiment which used many of the species in the current experiment, Mason et al. [25] found that native shrubs were significant competitors with bitou (as expressed by below ground biomass) under non-droughted conditions. Shrub species were also significantly more competitive against bitou than grass species. Functional groups based on growth form are easily recognisable and have potential to improve the generality and applicability of findings for conservation managers and practitioners alike.

Functional group effects on invasion are often considered similar to species effects [26]. However, whether species richness results scale up to functional richness is less clear. For example, Clark et al. [27] found that light capture in experimental grasslands was predicted by species richness better than functional diversity metrics. In addition, there is evidence against niche overlap as a mechanism of invader resistance at the functional level (e.g. [28, 29]).

We used a mesocosm experiment of constructed dune shrubland communities to examine the effect of functional richness and identity on invasibility, and on abiotic resource availability in the communities. We previously used a mesocosm approach to investigate the importance of arrival order on invasion success [4]. We found that arrival order of native functional groups was unimportant in invasion success. The mesocosms which make up two treatments (Unplanted and the grass, herb and shrub (GHS) multi functional group (FG) treatment), are shared by the arrival order and current study. While the previous study investigated the effect of different arrival orders and held functional richness constant in the planted treatments, the current study investigates the effect of functional richness and identity and holds arrival order constant. We used bitou (*Chrysanthemoides monilifera* ssp. *rotundata*) as our system invader. Bitou is a South African shrub species that has invaded large areas of coastal eastern Australia. It has been the subject of intensive management by practitioners but activities have often focused on reducing biomass rather than establishing functionally diverse native communities after control [30]. While bitou now dominates considerable areas of fore and hind dune communities, the resistance of native communities has seldom been tested. Historically bitou was actively planted as a stabiliser and has therefore experienced assisted dispersal [31]. Further, the invasion process has been assisted by both exogenous (mining, erosion caused by vehicles) and endogenous (storm, wave or wind erosive forces) disturbance regimes [31]. It is possible that bitou has responded to available resources rather than actively displacing native functional groups. Indeed, previous research has indicated that native communities have some resistance to bitou invasion. A study comparing sparsely- and heavily-invaded dune sites found that bitou seed availability alone does not determine the degree of invasion because bitou seed was represented in the sparsely-invaded seed banks [32]. We therefore infer that native communities themselves may be moderating bitou invasion by suppressing bitou germination.

We used an experimental mesocosm approach because it is difficult to control for variable historical disturbance regimes of natural settings. The experiment simulated post-control conditions: where bitou is cleared or standing dead biomass is left in situ, native species may be planted and bitou reinvasion can occur via seed from the seedbank or adjacent areas. Functional richness and identity varied across our constructed communities, while species richness and number of individuals along with exogenous factors of disturbance and fertilisation were held constant. This is important as the design of some diversity-invasibility studies means they are unable to disentangle the relative effects of species and functional group richness (e.g. [33, 34]).

We predicted that:

1. Communities with higher functional richness would be less invaded than those with lower functional richness;
2. Abiotic resource availability would be lowest in the most functionally rich planted treatment, highest in the unplanted treatment and intermediate for the low and moderate functional richness treatments;
3. Communities represented by functional groups similar to the invader would be less invaded than communities where all functional groups were distinct from the invader.

## Materials and Methods

### Experimental design

The mesocosm experiment was conducted between June 2007 and May 2009 at the University of Wollongong Shoalhaven Campus, New South Wales (34°53’18”S, 150°33’56”E) at a fenced outdoor facility. Details of the design are available in Mason et al. [4]. Briefly, each mesocosm was established in galvanised iron tanks (2.1 m diameter and 1.2 m high) with a drainage outlet at the base. We focused on plant interactions at this small scale to understand how resource availability influences invasion success [6]. Mesocosms were separated from neighbours by at least 1.5 m and filled with unwashed dune sand to a depth of 1 m. In June 2007 we introduced dune soil fauna via a soil inoculum and plant nutrients via a nitrogen/phosphorus/potassium (NPK) controlled release fertiliser with trace elements (250 g of Osmocote Native per 3.5 m^3^ of sand) in each tank. The fertiliser addition was repeated in April 2008. We measured total nitrogen (as total Kjeldahl nitrogen (TKN)) in October 2007 to allow soil chemistry to re-equilibrate after the setup disturbance and to allow release of the fertiliser. Resultant average soil nitrogen concentrations were low (0.002–0.024 mg TKN/g) and comparable to fore dune conditions [35].

We created 40 mesocosms which had constant species richness and number of individuals but which varied in functional richness and identity (Table 1). In cases where the functional richness was the same among different diversity treatments (Grass (G), Herb (H), Shrub (S) single functional group treatments (single FG) and GH, GS, HS multi functional group treatments (multi FG)), functional identity was varied. We removed volunteer colonisers (native and exotic) as they germinated in both planted and unplanted mesocosms, to retain experimental functional richness and identity values. There were five replicates for each treatment. Assemblages were comprised of species which commonly co-occur in coastal dune shrublands of eastern Australia and belong to three functional groups: rhizomatous or stoloniferous grasses, prostrate herbs and shrubs (Table 2). Species nomenclature followed Harden [36–39]. We avoided known nitrogen fixers during species selection. Each planted mesocosm consisted of six species, with four individuals planted for each species (24 individuals per mesocosm), and our species richness per unit area was similar to values for undisturbed fore dune shrublands (Mason unpublished data). Due to glasshouse and germination restrictions, we had a pool of six species representing each functional group. This meant that multi FG treatment replicates were comprised of species selected at random from the available pool. However, single FG treatments had the full complement of available species in each replicate, so our findings pertaining to the single FG treatments are particular to the species represented in this study. We were able to decouple species and functional richness effects because the number of species remains constant despite changing functional richness.

**Table 1:** Planting design denoting species and functional richness values used to construct experimental communities, where G = grass, H = herb, S = shrub.

Treatment	Experimental community composition*	Species richness	Functional richness
Unplanted**		0	0
G	G_1_, G_2_, G_3_, G_4_, G_5_, G_6_	6	1
H	H_1_, H_2_, H_3_, H_4_, H_5_, H_6_	6	1
S	S_1_, S_2_, S_3_, S_4_, S_5_, S_6_	6	1
GH	G_x_, G_y_, G_z_, H_x_, H_y_, H_z_	6	2
GS	G_x_, G_y_, G_z_, S_x_, S_y_, S_z_	6	2
HS	H_x_, H_y_, H_z_, S_x_, S_y_, S_z_	6	2
GHS	G_x_, G_y_, H_x_, H_y_, S_x_, S_y_	6	3

* an “x”, “y” or “z” subscript denotes a species drawn at random without replacement from the pool of six species comprising the functional group.

**while the unplanted treatment was not planted with native functional groups, it was invaded with bitou following the invasion event.

**Table 2:** Species pool used in experimental communities.

Functional group	Species
Rhizomatous / stoloniferous grasses	*Cynodon dactylon*
	*Digitaria didactyla*
	*Paspalum vaginatum*
	*Spinifex sericeus*
	*Sporobolus virginicus* var *virginicus*
	*Zoysia macrantha*
Prostrate herbs	*Calystegia soldanella*
	*Carpobrotus glaucescens*
	*Hydrocotyle peduncularis*
	*Melanthera biflora*
	*Tetragonia tetragonoides*
	*Viola hederacea*
Shrubs	*Breynia oblongifolia*
	*Correa alba*
	*Leptospermum laevigatum*
	*Myoporum boninense*
	*Rhagodia candolleana*
	*Westringia fruticosa*

Most species were grown from cuttings or division and sourced from coastal sites. One grass (*Spinifex sericeus*), one herb (*Tetragonia tetragonoides*) and two shrub (*Leptospermum laevigatum* and *Breynia oblongifolia*), species were grown from seed. The shrub species grown from seed were slow to germinate or grow and were given a lead time of 9–11 months prior to establishment of the rest of the species. All individuals were grown in 75 mm forestry tubes and were grown for at least two months prior to transplantation. Mesocosms were subject to ambient weather conditions, but we provided supplementary watering at the beginning, and during dry periods throughout the experiment. We ensured all mesocosms received the same water resources by timed application at a set hose pressure. We monitored plantings and replaced dead individuals as required for 11 months after planting. We then monitored mesocosms for species representation only, because the spreading habit of some grass and herb species made it difficult to identify individuals. The location of individuals in the mesocosm was assigned at random and individuals were planted equidistantly from each other and the mesocosm boundary.

We simulated an invasion event in January 2008; seven months after the mesocosms were planted with their native communities. Consequently, native individuals were at least nine months old at this time. We sowed approximately 1250 bitou seeds by weight in each mesocosm. This sowing rate was based on the viable seed bank of fore dune communities which had been invaded by a bitou monoculture, cleared and left fallow for two months (see [4] for further details), and our experiment should reflect the invasion potential of dune communities undergoing restoration. We collected bitou seed from a number of coastal sites. Prior to sowing, we tested seed viability (using tetrazolium chloride) on a subsample and adjusted our sowing rate to achieve the required viable seed bank density.

Biotic data include the influence of both the planted native community and the invader (data are taken from the invasion event onwards). Abiotic data reflect conditions immediately prior to the invasion event and then chart the influence of both the planted native community and the invader.

### Data collection

#### Biotic measurements

We measured bitou invasion success by counting the number of bitou individuals at intervals throughout the experiment (initially weekly, then fortnightly, then monthly as germination rates slowed). We also measured bitou cover once individuals became established (262 days after bitou sowing). Cover was measured by estimating percent bitou foliage cover in each mesocosm. At the end of the experiment, we harvested bitou individuals at ground level and bagged and dried the biomass. All biomass was dried at 60°C to a constant weight (±0.001 g).

We measured native functional group biomass by identifying and harvesting individuals at ground level, bagging and drying the biomass at 60°C to a constant weight (±0.001 g). We also sampled below ground biomass in each mesocosm, however, this biomass could not be differentiated into functional groups or native vs. bitou biomass. We used an hydraulic vibracorer to extract cores with a length of 90 cm and diameter of 7.4 cm (see [4] for further details). Three cores per mesocosm were extracted at the end of the experiment during February and March 2009. Cores were sectioned to reflect root stratification (0–20 cm; 20–50 cm; 50–75 cm; 75–90 cm) and we adjusted section lengths to account for compaction. Root material was extracted by washing, sieving (2 mm) and drying. The average mass per core section was recorded for each mesocosm (±0.001 g).

#### Abiotic measurement: Photosynthetically active radiation (PAR)

We took 20 random PAR measurements at ground level which were stratified among four quadrants in each mesocosm. We also took six PAR measurements above the canopy and calculated average percent light penetration to ground level. We measured PAR using Quantum sensors (Skye Instruments, Wales, UK; Li-Cor Environmental, Nebraska, USA). Measurements were initially taken each month (Jan-Aug 2008, except for July 2008 due to cloudy conditions) and then once every second month until the end of the experiment. Measurements were taken on clear days between 1000 and 1400 hours. Due to time constraints, PAR was only measured in the unplanted treatment at the end of the experiment. At the final time period, we further analysed PAR by developing light categories which were delineated by the compensation point (<50 μmol m^-2^s^-1^) and the saturation point (>1000 μmol m^-2^s^-1^) for species planted in the experiment. These categories were based on light response curves for two related species, *Paspalum dilatatum* [40] and *Paspalum notatum* [41] and corresponded to suboptimal (0–50 μmol m^-2^s^-1^), optimal (50–1000 μmol m^-2^s^-1^) and potentially supra-optimal (>1000 μmol m^-2^s^-1^) light for photosynthesis.

#### Abiotic measurement: Soil moisture

We measured moisture along the soil profile by auguring samples at depths of 0, 20, 50, 75 and 100 cm at five random locations stratified to include each quadrant of the mesocosm. Samples were bulked at each depth and stored in airtight plastic containers. We sampled at intervals through the experiment (December 2007 (pre bitou sowing), June 2008 (Day 150) and December 2008 (Day 338)) and used the gravimetric method to measure soil moisture: mass loss was determined after drying 5–10 g of soil at 60°C for at least 72 h.

#### Abiotic measurement: Soil temperature

We measured soil temperature just below the surface (2 cm) as this region is where fluctuations are most evident (e.g. [42]). We used five temperature sensors (D21921G-F5 Dallas Thermochron iButton) in two replicate meosocoms of each treatment. We placed each sensor at a random location stratified to include each quadrant of the mesocosm. The sensors measured temperature hourly for a month. They were then retrieved, the data downloaded and the sensors were reburied the following month. This data collection was continued for the duration of the experiment (December 2007 –December 2008) and new replicate mesocosms were chosen at random. We extracted average monthly minimum and maximum temperatures for analysis.

#### Abiotic measurement: Nutrients in soil leachate

We collected a leachate sample (1 L) from the base drainage outlet of each mesocosm in December 2007 (pre bitou sowing) and February (Day 46), May (Day 129), September (Day 243) and December 2008 (Day 338). Conditions were too dry in February 2009 to obtain sufficient leachate. We assumed that the dissolved soil nutrient leaching concentrations below the root zone were positively related to plant soil nutrient availability. We measured dissolved concentrations of Ba, Ca, Fe, K, Mg, Mo, Ni and P by inductively coupled argon plasma-atomic emission spectrometry (ICP-AES) (Spectroflame EOP, Spectro Analytical Instruments) [43]. Phosphorus concentrations were below detection limits (~100μg P L^-1^).

#### Abiotic measurement: Total soil nitrogen

We measured total nitrogen of dry soil by Kjeldahl analysis with SeSO_4_-K_2_SO_4_ catalyst using the method of Eaton et al. [44] with an ammonia selective electrode (Thermo Orion 9512) and a Thermo Orion meter (Model 720). Soil samples were obtained by bulking and mixing five random samples collected at 20 cm depth from each mesocosm (~20 g). Samples were collected in October 2007 (pre bitou sowing) and December 2008 (Day 338) only, due to time and laboratory constraints.

#### Abiotic measurement: Bare ground

We estimated bare ground cover in each mesocosm by estimating the vertical projection of foliage and branches [45] and then subtracting this value from 100 to give percent bare ground cover. Measurements were taken each month between February 2008 and January 2009.

### Statistical analyses

We used repeated measures ANOVA to analyse the effects of functional richness treatment and time on both biotic (number of bitou individuals, bitou cover) and abiotic variables (soil moisture and temperature, PAR, nutrient concentrations in leachate, bare ground cover). We used Pillai’s Trace multivariate test as it is robust to deviations from normality and homogeneity of variance [46]. A significant interaction term (time x functional richness treatment) necessitated analysis of functional richness effects for individual time periods. A non-significant interaction term allowed analysis of the effect of functional richness averaged over all time periods. A significant treatment effect was analysed with Tukey post hoc tests to identify differences among treatments. Where transformation did not improve homogeneity of variance, we used Welch’s test [47, 48] and Dunnett’s T3 post hoc test [47]. We performed analyses for biotic variables both with and without unplanted mesocosms included to determine if it was the unplanted treatment alone which drove functional richness relationships.

We analysed above ground native, bitou and total biomass collected at the final harvest using one factor ANOVAs. Similarly we analysed total nitrogen during the early and final stages of the experiment using one factor ANOVAs (functional richness treatment as the single factor in all cases). We used a two factor ANOVA (functional richness treatment and core depth as the two factors) to analyse below ground biomass at different core depths for the final harvest. We also used ANCOVA to examine whether a linear relationship existed between bitou biomass (dependent variable) and native biomass (covariate) at the final harvest. We wanted to test whether the relationship was similar across functional richness treatments.

We analysed PAR data at the final time period using a Chi-square test with a matrix of eight rows (functional richness treatments) and three columns (PAR categories). We used the adjusted residuals to identify treatments that contribute significantly to the overall Chi-square value (adjusted residual values outside ± 1.96 indicated cells departing most from independence [49]).

We transformed data where necessary to meet the assumptions of ANOVA and ANCOVA. Data were analysed using SPSS v. 17 software (SPSS Chicago IL, USA), and we present data as means with 95% confidence intervals.

## Results

### Invasion resistance

**Fig 1:**
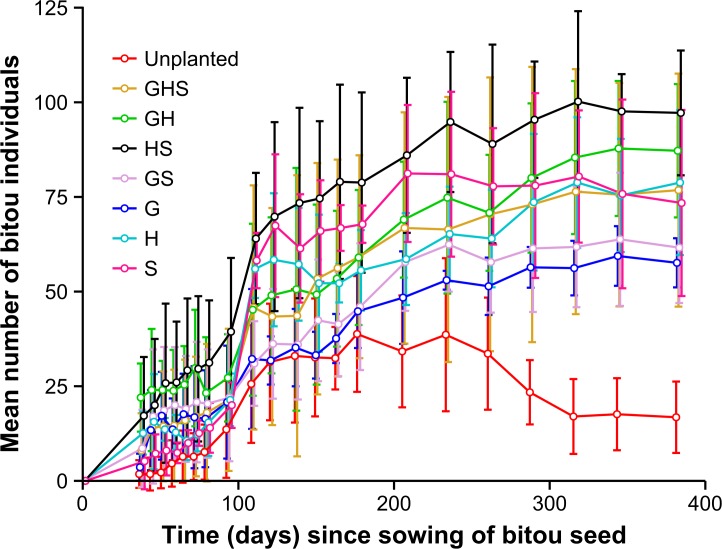
Mean number of bitou individuals for treatments over the sampling period. Error bars are 95% confidence intervals (*n* = 5). Unplanted = unplanted control; GHS = grass, herb and shrub multi FG treatment; GH = grass and herb multi FG treatment; HS = herb and shrub multi FG treatment; GS = grass and shrub multi FG treatment; G = grass single FG treatment; H = herb single FG treatment; S = shrub single FG treatment. See Methods for detailed description of treatments.

Differences in functional richness and identity among the constructed native communities did not strongly affect invasion resistance. A repeated measures ANOVA including the planted and unplanted treatments revealed a significant interaction between treatment and time for the number of bitou seedlings (*F*_140, 133_ = 1.50; *P* = 0.01; Fig 1). This interaction was driven by different responses by the planted and unplanted treatments because when the unplanted treatment was removed from the analysis, the interaction was non-significant (*F*_120, 84_ = 1.26; *P* = 0.13). When we examined effects among planted treatments, we found a trend towards differences in invasion success (*F*_6, 28_ = 2.31; *P* = 0.06; Fig 1). The Tukey test indicated a trend for lower invasion by bitou individuals in the grass single FG treatment compared with the HS multi FG treatment (*P* = 0.06) only. In any case, we found that bitou seedlings were able to germinate and establish for all levels of functional richness and identity.

**Fig 2:**
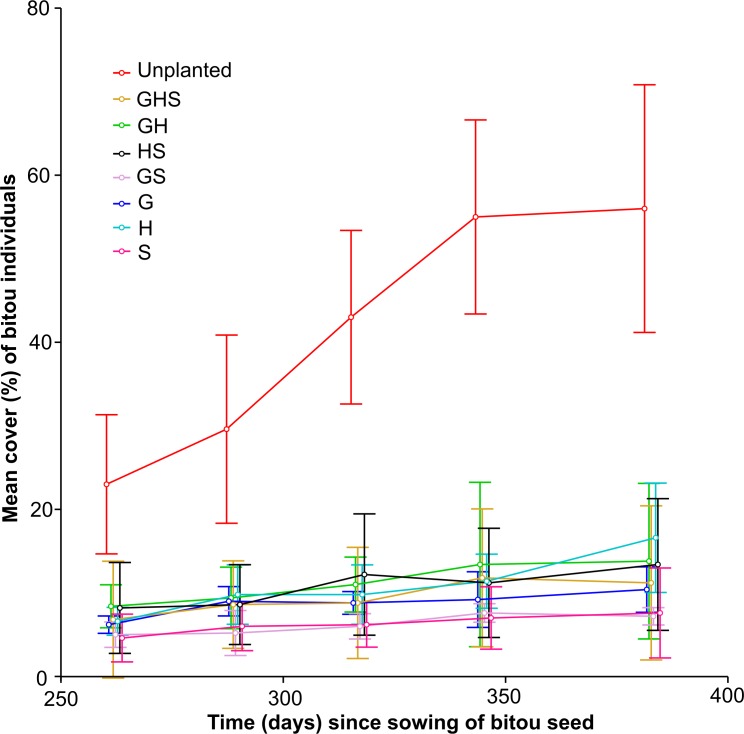
Mean percent of bitou cover for treatments over the sampling period. Error bars are 95% confidence intervals (*n* = 5).

A repeated measures ANOVA for bitou percent cover over time indicated a significant interaction between treatment and time (*F*_28, 128_ = 2.07; *P* < 0.01; Fig 2). The interaction was clearly driven by bitou cover increasing at an increasing rate (between 262 and 345 days) in the unplanted treatment (Fig 2). At the end of the experiment (Day 383), bitou cover was significantly higher in the unplanted treatment than all planted treatments, and the planted treatments had similarly low bitou cover levels (post hoc test results not shown). This pattern agrees with findings for final bitou above ground biomass. A one factor ANOVA indicated significant differences among treatments (*F*_7, 32_ = 22.53; *P* < 0.01), and post hoc test results indicated that bitou biomass was significantly higher in the unplanted treatment (mean ± 95% confidence intervals: 1901 ± 718 g) than all planted treatments, which had similar low values to each other (mean ± 95% confidence intervals: G: 146 ± 62 g; H: 219 ± 105 g; S: 69 ± 94 g; GH: 249 ± 296 g; GS: 80 ± 41 g; HS: 230 ± 218 g; GHS: 188 ± 276 g).

The significant interaction (time x functional richness treatment) was retained when the unplanted treatment was excluded from the analysis (*F*_24, 112_ = 1.75; *P* = 0.03). This interaction was driven by bitou cover remaining low and stable in GHS (*F*_4, 20_ = 0.84; *P* = 0.52), GH (*F*_4, 20_ = 0.89; *P* = 0.49) and HS (*F*_4, 20_ = 0.94; *P* = 0.46) multi FG treatments and the shrub single FG treatment (S: *F*_4, 20_ = 1.03; *P* = 0.42). But bitou cover increased over time for the grass and herb single FG treatments (G: *F*_4,20_ = 3.73, *P* = 0.02; Tukey test: *P* = 0.01; Day 262 < Day 383) (H: *F*_4,20_ = 5.72, *P* <0.01; Tukey test: *P* <0.01; Day 262 < Day 383) and GS multi FG treatments (*F*_4,20_ = 3.58, *P* = 0.02; however post hoc tests could not differentiate between time periods indicating that the effect was not strong).

When looking specifically at the Unplanted treatment, the hump-shaped (unimodal) response of bitou individuals over time (Fig 1), along with increases in bitou cover (Fig 2), may indicate self-thinning by bitou in the latter stages of the experiment. To validate the unimodal pattern in bitou individuals for the Unplanted treatment (Fig 1), we compared the mean number of bitou individuals in the early stage of the experiment (t = 94 days) to that at the end (t = 383 days) and found a similar number of bitou individuals (*F*_1,8_ = 0.31; *P* = 0.59). However, during the middle stage of the experiment (t = 207 days), the number of individuals was significantly higher than in the early stage and the end (t = 94 days < t = 207 days (*F*_1,8_ = 8.56; *P* = 0.02) and t = 207 days > t = 383 days (*F*_1,8_ = 7.59; *P* = 0.03). In contrast, bitou individuals increased to a relatively stable level for most planted functional groups (individuals at t = 207 days < individuals at t = 383 days for the herb (H) treatment (*F*_1,8_ = 6.11; *P* = 0.04), but *P* > 0.05 for all other planted functional groups). This response may indicate facilitation of bitou by native functional groups in planted treatments.

### Final native, bitou and total above ground biomass across treatments

**Fig 3:**
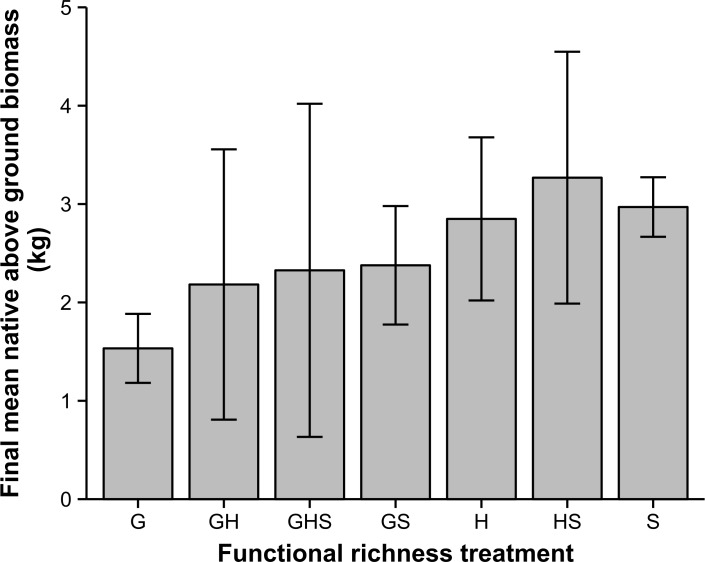
Final mean native above ground biomass (kg) across different treatments. Error bars are 95% confidence intervals (*n* = 5).

Final native above ground biomass did not have a significant response to functional richness treatments (*F*_6,28_ = 2.37, *P* = 0.06), although there was a trend of reduced biomass for treatments which included the grass functional group (Fig 3).

The ANCOVA (which excluded the Unplanted treatment because it did not have any native biomass), had a non-significant interaction term (*F*_*6*,*21*_ = 0.59; *P* = 0.74). This result indicated similar slopes of regression lines among planted treatments. Of the main effects, final native above ground biomass had a significant negative relationship with bitou biomass (*F*_*1*,*21*_ = 10.62; *P* < 0.01), however the functional richness treatment was non-significant (*F*_*6*,*21*_ = 0.76; *P* = 0.61).

Final total above ground biomass (native and bitou biomass) was significantly different across treatments (*F*_7,32_ = 3.42; *P* = 0.01). Post hoc tests indicated that Unplanted and G treatments had significantly lower total biomass than HS.

### Final below ground biomass across treatments

**Fig 4:**
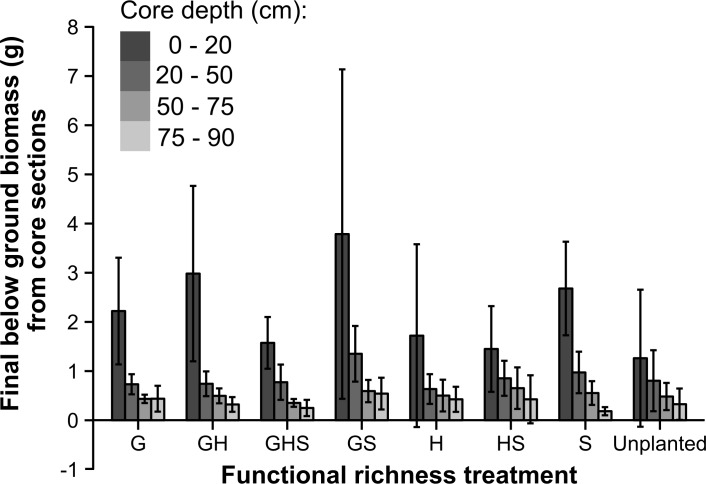
Final mean below ground biomass (g) for each functional richness treatment at different soil depths. Error bars are 95% confidence intervals (*n* = 5).

There was a significant interaction between functional richness treatment and core depth in a two factor ANOVA (*F*_21,128_ = 1.86; *P* = 0.02). As expected, the zone closest to the soil surface supported the highest root biomass at the end of the experiment, but the significant interaction was driven by different magnitudes in root biomass close to the soil surface across different treatments (Fig 4). A one factor ANOVA comparing functional richness treatments at the top of the soil profile indicated a significant treatment effect (*F*_7,32_ = 3.01; *P* = 0.02). But the only significant difference in biomass close to the soil surface was due to the unplanted treatment supporting significantly less root biomass than the GS treatment (*P* = 0.03).

### Abiotic response variables

Response patterns for average monthly minimum and maximum soil temperatures were consistent for all treatments over time (non-significant interaction between time and treatment). However, there were significant overall treatment effects (Table 3). We found evidence of an insulating effect of the grass functional group. Unplanted mesocosms experienced lower minimum soil temperatures than all grass multi FG treatments (GH, GS, GHS), and the grass single FG treatment (G), but minimum temperatures in unplanted mesocosms were similar to the herb and shrub single FG (H, S) treatments along with the HS multi FG treatment. There was some evidence of an insulating effect of the grass (G) treatment compared with the other single FG and the HS multi FG treatment (See S1 Table).

**Table 3:** Results of repeated measures ANOVA for effects of functional diversity on abiotic resource availability variables.

	Pillai’s trace multivariate test (time x treatment)	Between treatment effects
**Soil temperature**		
Average monthly minimum	*F*_42,48_ = 0.94; *P* = 0.58	***F***_**7,8**_ **= 10.83; *P* < 0.01**
Average monthly maximum	*F*_42,48_ = 1.43; *P* = 0.11	***F***_**7,8**_ **= 7.57; *P* < 0.01**
**Soil moisture**		
0 cm	*F*_14,54_ = 1.66; *P* = 0.09	*F*_7,27_ = 0.67; *P* = 0.70
20 cm	***F***_**14,54**_ **= 2.65; *P* = 0.01**	n/a
50 cm	***F***_**14,54**_ **= 2.44; *P* = 0.01**	n/a
75 cm	***F***_**14,54**_ **= 3.62; *P* <0.01**	n/a
100 cm	*F*_14,54_ = 1.16; *P* = 0.34	*F*_7,27_ = 1.72; *P* = 0.15
**Photosynthetically active radiation at ground level**^a^		
	*F*_48,156_ = 1.43; *P* = 0.06	*F*_6,28_ = 1.76; *P* = 0.15
**Bare ground cover (%)**		
	*F*_77,196_ = 1.12; *P* = 0.26	***F***_**7,32**_ **= 43.71; *P* < 0.01**
**Nutrient concentration in soil leachate**		
Barium	***F***_**21,84**_ **= 3.43; *P* < 0.01**	n/a
Calcium	*F*_28,80_ = 0.81; *P* = 0.73	*F*_7,20_ = 0.75; *P* = 0.63
Iron	***F***_**21,78**_ **= 1.71; *P* = 0.05**	n/a
Magnesium	*F*_21,78_ = 1.60; *P* = 0.07	***F***_**7,26**_ **= 6.67; *P* < 0.01**
Molybdenum	***F***_**21,84**_ **= 2.22; *P* < 0.01**	n/a
Nickel	***F***_**21,84**_ **= 2.78; *P* < 0.01**	n/a
Potassium	***F***_**28,80**_ **= 2.37; *P* < 0.01**	n/a

a Measured for planted treatments only (i.e. excluding unplanted treatment); n/a not applicable

We found further evidence of insulating effects of the grass functional group and potential non-insulating effects of the shrub functional group when examining the treatment effects for average maximum soil temperature. Post hoc test results indicated that soil just below the surface in the grass (G) treatment was cooler than the Unplanted, HS and S treatments. The GH multi FG also had soil temperatures which were cooler than the HS and S treatments (See S1 Table). The moderating effect of the G and GH treatments was not evident for the GS and GHS multi FG treatments, and it may be that the elevated biomass characteristic of the shrub functional group counteracts the insulating properties of grasses in these constructed communities.

Soil moisture at 20, 50 and 75 cm was significantly different over time (significant time x treatment interactions, Table 3). We therefore examined responses at each time period. Welch’s test was significant at a depth of 20 cm for December 2007 (pre bitou sowing) (F_7,12.9_ = 3.79; *P* = 0.02), but post hoc tests were unable to distinguish between treatments. At a depth of 50 cm, Welch’s test was significant for December 2007 (pre bitou sowing) (F_7,12.9_ = 12.88; *P* < 0.01). Dunnett T3 test indicated that soil moisture in the Unplanted treatment was significantly higher than the HS treatment for this time period. At a depth of 75 cm, a one-way ANOVA was significant for December 2007 (F_7,32_ = 3.71; *P* = 0.01) and December 2008 (Day 338) (F_7,27_ = 4.25; *P* < 0.01). Tukey test results indicated that GHS and HS treatments had significantly lower soil moisture than the S treatment in December 2007, while H, S, GS, GH and GHS had significantly lower soil moisture than the Unplanted treatment in December 2008. Soil moisture at a depth of both 0 cm and 100 cm was consistent across time periods and treatment effects were non-significant (Table 3).

**Fig 5:**
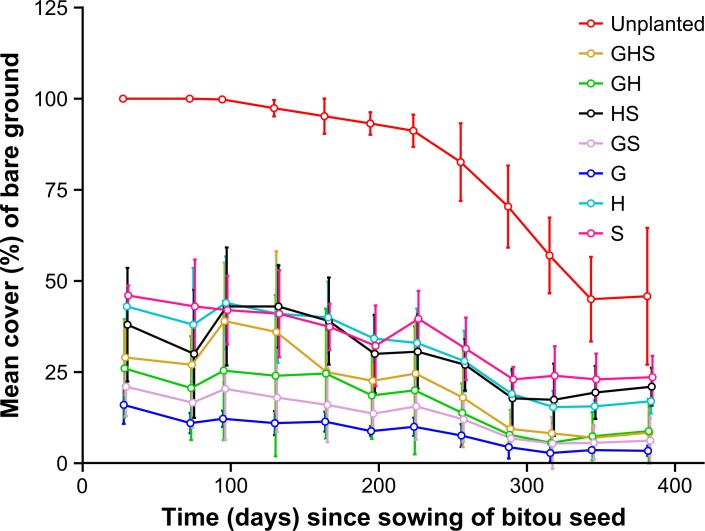
Mean bare ground cover (%) for treatments over the sampling period. Error bars are 95% confidence intervals (*n* = 5).

The patterns for bare ground cover were consistent over time for all treatments (non-significant time x treatment interaction), but there was a significant overall treatment effect (Table 3). The unplanted treatment had significantly higher bare ground cover than all planted treatments. Among the planted treatments, those which included the grass functional group tended to have lower bare ground cover than the other treatments (post hoc test results: G, GH, GS < H, HS, S; G < GHS < S; S1 Table; Fig 5).

Photosynthetically active radiation (PAR) at ground level was similar across planted treatments for the duration of the experiment (Table 3) and a one factor ANOVA at the final time period which included the unplanted treatment indicated a similar result (F_7,32_ = 0.99; *P* = 0.46). However, when light data were categorised into sub-optimal, optimal and supra-optimal PAR at the final time period, we found a significant association between PAR category and functional richness treatment (χ^2^_(14)_ = 37.22; *P* < 0.01). Adjusted residuals indicated that the unplanted and grass single FG treatments had significantly more, and the HS treatment had significantly fewer sub-optimal PAR counts than expected: the unplanted and grass (G) treatments had more deep shade than expected, presumably due to bitou canopy and grass cover respectively. In addition, the HS treatment had significantly more optimal PAR counts than expected, while the GH treatment had significantly more supra-optimal PAR counts than expected.

**Fig 6:**
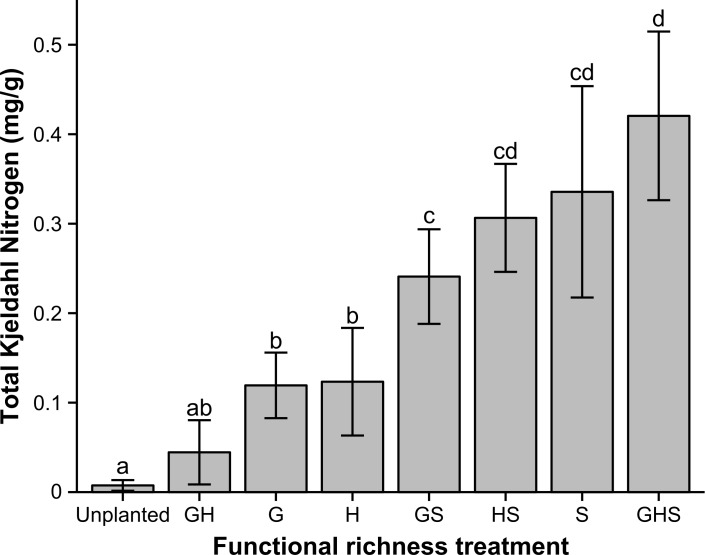
Mean total soil nitrogen (mg / g dry soil) for treatments in December 2008 (Day 338). Error bars are 95% confidence intervals (*n* = 5). Different letters indicate significant differences among treatments (*P* < 0.05).

Average total soil nitrogen (mg/g dry soil) was similarly low across all treatments when sampled in the first year of the experiment (October 2007 (pre bitou sowing): *F*_7,32_ = 1.62; *P* = 0.17). However, prior to biomass harvest, there were significant differences in soil nitrogen among treatments (December 2008 (Day 338): *F*_7,32_ = 44.38; *P* < 0.01). Post hoc analyses indicated that treatments which included the shrub functional group in the planting treatment had higher total soil nitrogen than those where the shrub functional group was not represented (S1 Table; Fig 6). Shrubs increased total soil nitrogen.

The concentration of nutrients in soil leachate over time appeared unrelated to functional richness treatments. The concentration of calcium over time did not vary among treatments and the treatment effect was non-significant (Table 3). The interaction term was non-significant for magnesium also, but there was a significant treatment effect (post hoc test results: Unplanted, GH, H, GS < S; Unplanted < GHS, HS; GS < GHS; S1 Table). This response appeared unrelated to functional richness. The other measured nutrients in the soil leachate displayed significant interactions between time and treatment (Table 3). However, when we analysed nutrient responses at each time period, they again appeared unrelated to functional richness (S2 Table).

## Discussion

### Functional richness does not mediate invasion resistance

Our study did not support the prediction that increased functional richness provides greater invasion resistance in native communities. Greater functional richness in the GHS treatment did not confer greater resistance in terms of either the number of invader individuals or invader cover. In fact, the only planted treatment to display a trend of suppressing bitou individuals was the grass treatment (G)–a treatment with the lowest functional richness. This trend of fewer bitou individuals in the grass treatment than other planted communities is supported by our results for photosynthetically active radiation (PAR) at ground level at the final time period; unplanted and grass single FG treatments had more suboptimal light patches than expected and the HS multi FG treatment had more optimal light patches than expected. Concordantly, the unplanted treatment had fewer bitou individuals than all other treatments, and the grass single FG treatment showed a trend of fewer bitou individuals than the HS multi FG treatment. So there was some evidence that light availability was affected by the functional richness treatment. Our results are consistent with other studies which have found a positive relationship between invasion and light availability (e.g. [50, 51]). Our results are also consistent with early rangeland observations where native grasses resisted woody invader encroachment [52]. More recently, a study by Fargione [16] found that native grasses suppress invaders from other functional groups by reducing soil nitrate availability. In our case, it appears that grasses reduce light resources for juvenile bitou individuals.

All planted treatments supported significantly more bitou individuals than the unplanted treatment. We attribute this pattern to a nurse effect among extant native species (e.g. [53]). Bare ground was significantly higher in unplanted than planted treatments and the exposed conditions of the unplanted treatment may reduce bitou germination and establishment. More generally, native functional groups may directly protect bitou seed from dislodgement following wind or rain action. Native plantings may also moderate microclimatic factors at the soil surface and facilitate bitou germination [54]. We did not find consistent evidence for microclimatic moderation in our abiotic resource results, but our measurement scales (spatial and temporal) may have been too coarse to observe these effects, especially in the immediate post-invasion environment.

Facilitation of invasives by native species is increasingly recognised as an important interaction in plant communities [55] and it may explain the poor relationship between functional group richness and invasibility in the current study. Facilitation of bitou by native functional groups may counter their competitive effects and explain the deviation from our prediction that invasibility will decline with increasing functional group richness [56].

However, we did not see facilitative effects of native functional groups when considering bitou cover: all native communities similarly suppressed bitou cover. Our findings agree with a meta-analysis of competition studies which reported that native species interactions regulate invader abundance rather than completely repel invaders [26].

We attribute our finding of similar invasibility among all functional richness treatments to a bulk plant biomass or coverage effect. Native plant biomass at the final time period was similar among all planted treatments, but there was a trend of reduced biomass in communities with grass representation (*P* = 0.06). There was a complementary pattern of reduced bare ground cover among communities with grass representation. So grassed communities may make up for reduced vertical biomass with increased horizontal coverage. Below ground biomass effects at the end of the experiment were modest: the only significant difference at the 0–20 cm level (where we expect strong resource competition) was greater biomass for the GS treatment compared with the unplanted treatment. These results combine to suggest that bulk above-ground plant cover (horizontal or vertical) may affect invasion levels more than functional group arrangement. This interpretation is supported by the negative relationship between native and bitou biomass at the final time period, and the finding that functional richness treatment does not affect the relationship between bitou biomass and native biomass (ANCOVA results with the Unplanted treatment removed). In a related mesocosm arrival order study, we found that functional identity and historical dominance were unimportant in determining invasion resistance [4]. We reached a similar conclusion that invasion may be responding to bulk plant biomass and above-ground net primary productivity rather than arrival order.

The experiment has identified two potentially counteracting invasion drivers: nurse effects of native biomass on bitou individuals and native bulk biomass resistance to bitou biomass accumulation. It is unclear which driver will be most important in long term vegetation dynamics. Longer term mesocosm or field experiments may provide clarity.

While diversity-invasibility studies have reported a negative relationship between functional richness and invasibility (e.g. [57, 58]), there have also been reports of neutral relationships (e.g. [19, 34]) which support our findings. Our study therefore argues against a general assembly rule that functional richness confers invasion resistance.

### Abiotic resource availability does not explain invasibility

Contrary to our second prediction, we found that abiotic resource availability did not vary consistently among the functional richness treatments. And resource availability in the unplanted treatment was often indistinguishable from planted treatments. For example, soil moisture levels and nutrient availability could not be explained by functional richness or the coarser metric of planted *vs*. unplanted treatments. This result may indicate that abiotic resources were not limiting in any of our communities: natives, the invader and different growth forms used abiotic resources in a similar, non-limiting way. Alternatively, bitou may be acting as an equaliser across treatments–allowing all functional richness treatments to optimise resource use. This argument is supported by final total above ground biomass (native and bitou biomass) results: the unplanted treatment had similar final total biomass to most planted treatments. The only significance we found was that unplanted and G communities had lower total biomass than the HS community.

It is noteworthy that there were discrepancies in the results for bare ground cover and PAR at ground level at the end of the experiment. Bare ground cover was significantly greater in the unplanted treatment (which supported the invader alone) than all planted treatments. However averaged PAR values for each mesocosm yielded similar values across all treatments, and when PAR was categorised into sub-optimal, optimal and supra-optimal values, the unplanted treatment had significantly more sub-optimal PAR counts (i.e. lower light penetration) than expected. So bare ground cover is not a direct proxy for PAR at ground level. These findings raise an unexpected methodological consideration for diversity studies which measure resource availability. Light interception is dependent on both total above ground biomass and the geometric configuration of the canopy [27], so using bare ground cover as a proxy for light availability, or attempting to summarise PAR by averaging across the community may overly simplify a complex process. Another study has highlighted the complexity of vegetation density and cover effects on light interception. It found that the effect of dicots on light levels in a grassland system was captured by percentage cover alone, but grass effects were expressed by a number of parameters (biomass, cover, height) [59]. We advocate categorisation of PAR based on light quality within each community prior to any comparison across treatments.

### Functional and invader identity effects

Contrary to our third prediction, inclusion of the shrub functional group in a community did not improve invasion resistance (number of bitou individuals or cover of bitou) relative to communities that did not have shrub representation. Our results therefore do not support the concept that greater niche overlap improves invader resistance (e.g. [12]). Our results are consistent with other research which challenges the importance of intra-guild structure in determining invasibility of plant communities (e.g. [28, 29]).

We observed greater intra- than inter-specific competition for the shrub invader as demonstrated by the hump-shaped response of bitou individuals over time in the unplanted treatment compared with the pattern of individuals increasing and then transitioning to a relatively stable level for most planted treatments. While these results demonstrate that competition may contribute to community structure, the biotic effects are modest with no significant effect on invasion resistance through niche pre-emption. It is interesting that intra-species competition was more important than inter-species competition for bitou individuals and may indicate that bitou is a strong invader [60].

Our results indicated that functional richness does not confer resistance. Our design controlled the invasion event, so we can exclude propagule pressure as an explanatory variable. And we have not found evidence for niche overlap as a significant factor in invasion resistance. We offer two possible explanations for the result that all our planted communities had similar resistance in terms of bitou cover and, to a lesser extent, number of bitou individuals. Firstly, invader identity may be more important than resident diversity in determining community resistance to invasion. Bitou may be a “strong” invader [60] and its ability to germinate and establish may overwhelm modest functional group or identity resistance effects. Other studies have also challenged the primacy of biotic resistance in controlling habitat invasibility [28, 33, 54, 61]. Secondly, species characteristics within a functional group may vary more than between functional groups [51] and this variation may obscure patterns. Our current results identify some functional group effects (e.g. fertility island effect of nitrogen for the shrub functional group (see [4] for potential explanation) and insulating effects associated with the grass functional group). And our previous research [25] has found differences in competitive effects among grass and shrub growth forms on the bitou invader. However, abiotic resource availability was generally similar among the defined functional groups. So we cannot exclude the possibility that, in this multispecies experiment, our selection of functional groups is largely indistinguishable in terms of resource use and invasion resistance. Another possibility is that an increased sample size would elucidate differences among functional groups.

We found that invasion resistance was unrelated to native functional group richness or identity in our constructed communities. Native constructed communities influenced the invader more strongly through suppression of adult biomass than through inhibition of seedling establishment. While the grass single FG treatment showed a trend of reducing bitou individuals relative to the HS multi FG treatment, in general all planted treatments had significantly more bitou individuals than the unplanted treatment, and this pattern remained consistent over time. Conversely, all planted treatments had significantly lower bitou cover (predominantly consisting of adult bitou biomass in the final stages of the experiment) than the unplanted treatment. These findings indicate that (1) extant native biomass significantly reduces invader biomass compared with bare ground; (2) increasing functional richness does not increase resistance to invasion; and (3) regardless of native functional richness, invader individuals are able to establish and potentially founder future populations following disturbance. Grass, herb and shrub growth forms dominate coastal fore dune communities. Our findings suggest that these growth forms have similar resource acquisition and may form a large single guild—at least in terms of invasion resistance. As a result, invasion outcomes in native communities may be dictated more by chance [1] and propagule pressure [62] than biotic effects. At the local level, bulk plant biomass rather than functional richness is more important in invasion resistance.

## Supporting Information

S1 File Datasets used for the analyses (XLSX)

S1 Table Significant post hoc results for abiotic variables (Bare ground cover (%), Average monthly minimum and maximum soil temperatures (°C), Final Total Kjeldahl Nitrogen and Magnesium concentrations in soil leachate) (DOCX)

S2 Table Single month ANOVA results for the effects of functional richness on soil nutrient availability (DOCX)
