# Supplementary material for: Functional Richness and Identity Do Not Strongly Affect Invasibility of Constructed Dune Communities
Source: PLoS One. 2017 Jan 10;12(1):e0169243. doi: 10.1371/journal.pone.0169243 (PMC5224978; doi:10.1371/journal.pone.0169243)
Supplement: S1 Table — (DOCX) [file pone.0169243.s002.docx]

**S1 Table. Significant post hoc results for abiotic variables (Bare ground cover (%), Average monthly minimum and maximum soil temperatures (⁰C), Final Total Kjeldahl Nitrogen and Magnesium concentrations in soil leachate): Multiple comparisons with Tukey tests for each treatment averaged across all time periods. Only differences that are significant at a family-wise error rate of 0.05 are shown**

| **Treatment comparisons** | | **Mean Difference** | **Significance** | **95% Confidence Interval** | |
| --- | --- | --- | --- | --- | --- |
|  |  |  |  | Lower Bound | Upper Bound |
| **Bare ground cover** | | | | | |
| Unplanted | G | 6.06 | <0.01 | 4.76 | 7.37 |
| Unplanted | GH | 5.02 | <0.01 | 3.72 | 6.33 |
| Unplanted | GHS | 4.54 | <0.01 | 3.24 | 5.84 |
| Unplanted | GS | 5.44 | <0.01 | 4.13 | 6.74 |
| Unplanted | H | 3.49 | <0.01 | 2.19 | 4.80 |
| Unplanted | HS | 3.58 | <0.01 | 2.28 | 4.89 |
| Unplanted | S | 3.17 | <0.01 | 1.86 | 4.47 |
| G | GHS | -1.52 | 0.01 | -2.83 | -0.22 |
| G | H | -2.57 | <0.01 | -3.87 | -1.27 |
| G | HS | -2.48 | <0.01 | -3.78 | -1.18 |
| G | S | -2.90 | <0.01 | -4.20 | -1.59 |
| GH | H | -1.53 | 0.01 | -2.83 | -0.23 |
| GH | HS | -1.44 | 0.02 | -2.74 | -0.14 |
| GH | S | -1.86 | <0.01 | -3.16 | -0.55 |
| GHS | S | -1.37 | 0.03 | -2.68 | -0.07 |
| GS | H | -1.94 | <0.01 | -3.24 | -0.64 |
| GS | HS | -1.85 | <0.01 | -3.16 | -0.55 |
| GS | S | -2.27 | <0.01 | -3.57 | -0.97 |
| **Average monthly minimum soil temperatures** | | | | | |
| Unplanted | G | -2.61 | <0.01 | -3.91 | -1.30 |
| Unplanted | GH | -1.34 | 0.04 | -2.65 | -0.03 |
| Unplanted | GHS | -1.58 | 0.02 | -2.89 | -0.27 |
| Unplanted | GS | -1.92 | <0.01 | -3.23 | -0.62 |
| G | H | 1.50 | 0.02 | 0.20 | 2.81 |
| G | HS | 1.48 | 0.03 | 0.17 | 2.78 |
| G | S | 1.76 | 0.01 | 0.45 | 3.06 |
| **Average monthly maximum soil temperatures** | | | | | |
| G | Unplanted | -2.82 | 0.04 | -5.46 | -0.18 |
| G | HS | -3.03 | 0.02 | -5.67 | -0.39 |
| G | S | -3.47 | 0.01 | -6.11 | -0.83 |
| GH | HS | -2.77 | 0.04 | -5.41 | -0.13 |
| GH | S | -3.21 | 0.02 | -5.86 | -0.57 |
|  | |  |  |  | |
| **Treatment comparisons** | | **Mean Difference** | **Significance** | **95% Confidence Interval** | |
|  |  |  |  | Lower Bound | Upper Bound |
| **Final Total Kjeldahl Nitrogen** | | | | | |
| Unplanted | G | -0.05 | <0.01 | -0.08 | -0.01 |
| Unplanted | GHS | -0.15 | <0.01 | -0.19 | -0.11 |
| Unplanted | GS | -0.09 | <0.01 | -0.13 | -0.05 |
| Unplanted | H | -0.05 | <0.01 | -0.08 | -0.01 |
| Unplanted | HS | -0.11 | <0.01 | -0.15 | -0.08 |
| Unplanted | S | -0.12 | <0.01 | -0.16 | -0.08 |
| G | GHS | -0.10 | <0.01 | -0.14 | -0.07 |
| G | GS | -0.04 | <0.01 | -0.08 | -0.01 |
| G | HS | -0.07 | <0.01 | -0.10 | -0.03 |
| G | S | -0.08 | <0.01 | -0.11 | -0.04 |
| GH | GHS | -0.13 | <0.01 | -0.17 | -0.10 |
| GH | GS | -0.07 | <0.01 | -0.11 | -0.04 |
| GH | HS | -0.10 | <0.01 | -0.13 | -0.06 |
| GH | S | -0.11 | <0.01 | -0.14 | -0.07 |
| GHS | GS | 0.06 | <0.01 | 0.02 | 0.10 |
| GHS | H | 0.10 | <0.01 | 0.07 | 0.14 |
| GS | H | 0.04 | 0.01 | 0.01 | 0.08 |
| H | HS | -0.07 | <0.01 | -0.10 | -0.03 |
| H | S | -0.07 | <0.01 | -0.11 | -0.04 |
| **Magnesium concentrations in soil leachate** | | | | | |
| Unplanted | GHS | -37.45 | <0.01 | -66.02 | -8.87 |
| Unplanted | HS | -37.15 | <0.01 | -67.27 | -7.02 |
| Unplanted | S | -48.48 | <0.01 | -81.01 | -15.94 |
| GH | S | -36.07 | 0.02 | -68.61 | -3.54 |
| GHS | GS | 30.23 | 0.03 | 1.65 | 58.80 |
| GS | S | -41.26 | <0.01 | -73.80 | -8.73 |
| H | S | -35.39 | 0.02 | -66.50 | -4.28 |
