# Supplementary material for: Functional Richness and Identity Do Not Strongly Affect Invasibility of Constructed Dune Communities
Source: PLoS One. 2017 Jan 10;12(1):e0169243. doi: 10.1371/journal.pone.0169243 (PMC5224978; doi:10.1371/journal.pone.0169243)
Supplement: S2 Table — (DOCX) [file pone.0169243.s003.docx]

**S2 Table. Single month ANOVA results for the effects of functional richness on soil nutrient availability**

| **Nutrient** | **Time period** | **Days since sowing bitou seed** | **Welch test** | **Dunnett’s T3*** |
| --- | --- | --- | --- | --- |
| Ba | **Dec 2007** | **Prior to sowing** | **F_7,13.6_ = 127.41; *P* < 0.01** | **S, H < HS < GS, GH, Unplanted; HS < GHS < GS** |
|  | Feb 2008 | 46 | F_7,13.6_ = 1.69; *P* = 0.19 |  |
|  | May 2008 | 129 | F_7,12.9_ = 2.19; *P* = 0.11 |  |
|  | **Sept 2008** | **243** | **F_7,12.1_ = 3.09; *P* = 0.04** | **Unplanted < H** |
| Fe | Dec 2007 | Prior to sowing | F_7,13.5_ = 2.15; *P* = 0.11 |  |
|  | Feb 2008 | 46 | F_7,13.6_ = 0.70; *P* = 0.67 |  |
|  | **May 2008** | **129** | **F_7,12.1_ = 3.43; *P* = 0.03** | *P* > 0.05 for all comparisons |
|  | Sept 2008 | 243 | F_7,12.0_ = 2.22; *P* = 0.11 |  |
| Mo | **Dec 2007** | **Prior to sowing** | **F_7,13.2_ = 38.24; *P* < 0.01** | **GH < H, S, HS < GHS** |
|  | **Feb 2008** | **46** | **F_7,12.6_ = 4.46; *P* = 0.01** | **G < GHS, HS, H, S** |
|  | May 2008 | 129 | F_7,12.4_ = 0.88; *P* = 0.55 |  |
|  | **Sept 2008** | **243** | **F_7,12.2_ = 2.93; *P* = 0.05** | *P* > 0.05 for all comparisons |
| Ni | **Dec 2007** | **Prior to sowing** | **F_7,13.6_ = 8.83; *P* < 0.01** | **G < GHS, HS, S** |
|  | **Feb 2008** | **46** | **F_7,13.5_ = 3.41; *P* = 0.03** | *P* > 0.05 for all comparisons |
|  | **May 2008** | **129** | **F_7,12.7_ = 3.34; *P* = 0.03** | **S < Unplanted** |
|  | Sept 2008 | 243 | F_7,12.2_ = 1.74; *P* = 0.19 |  |
| K | Dec 2007 | Prior to sowing | F_7,13.4_ = 0.78; *P* = 0.62 |  |
|  | **Feb 2008** | **46** | **F_7,13.3_ = 5.12; *P* < 0.01** | **H<Unplanted** |
|  | **May 2008** | **129** | **F_7,12.2_ = 4.84; *P* < 0.01** | **H, G < S** |
|  | **Sept 2008** | **243** | **F_7,11.6_ = 9.68; *P* <0.01** | **GS, HS < G, GH** |
|  | **Dec 2008** | **338** | **F_7,8.8_ = 13.50; *P* <0.01** | **HS < G, GH; S < G** |

* significant comparisons are indicated at *P* < 0.05.
